# ZENBU-Reports: a graphical web-portal builder for interactive visualization and dissemination of genome-scale data

**DOI:** 10.1093/nargab/lqad075

**Published:** 2023-08-21

**Authors:** Jessica Severin, Saumya Agrawal, Jordan A Ramilowski, Ruslan Deviatiiarov, Jay W Shin, Piero Carninci, Michiel de Hoon

**Affiliations:** RIKEN, Center for Integrative Medical Sciences, Yokohama, 230-0045, Japan; RIKEN, Center for Integrative Medical Sciences, Yokohama, 230-0045, Japan; RIKEN, Center for Integrative Medical Sciences, Yokohama, 230-0045, Japan; Advanced Medical Research Center, Yokohama City University, Yokohama, Japan; International Research Center for Medical Sciences, Kumamoto University, Kumamoto, Japan; RIKEN, Center for Integrative Medical Sciences, Yokohama, 230-0045, Japan; Genome Institute of Singapore, Agency for Science, Technology and Research (A*STAR), Singapore, Singapore; RIKEN, Center for Integrative Medical Sciences, Yokohama, 230-0045, Japan; Human Technopole, Milan, Italy; RIKEN, Center for Integrative Medical Sciences, Yokohama, 230-0045, Japan

## Abstract

In the genomic era, data dissemination and visualization is an integral part of scientific publications and research projects involving international consortia producing massive genome-wide data sets, intra-organizational collaborations, or individual labs. However, creating custom supporting websites is oftentimes impractical due to the required programming effort, web server infrastructure, and data storage facilities, as well as the long-term maintenance burden. ZENBU-Reports (https://fantom.gsc.riken.jp/zenbu/reports) is a web application to create interactive scientific web portals by using graphical interfaces while providing storage and secured collaborative sharing for data uploaded by users. ZENBU-Reports provides the scientific visualization elements commonly used in supplementary websites, publications and presentations, presenting a complete solution for the interactive display and dissemination of data and analysis results during the full lifespan of a scientific project both during the active research phase and after publication of the results.

## INTRODUCTION

Scientific publications are often accompanied by a dedicated website that manages and organizes the data, showcases the analysis results, and offers analysis tools to interact with the data. Such accompanying websites are important both for consortia projects that produce massive genome-wide data sets, and increasingly also for smaller projects with limited resources. Usually, these supporting websites are custom-constructed manually for each publication, requiring the services of a software engineer, web server infrastructure with large data storage capacity, as well as continuous software maintenance.

ZENBU-Reports builds on the ZENBU Genome Browser system (1), created as part of the FANTOM5 Project (2,3) to visualize large-scale next-generation sequencing data mapped to a genome. In contrast, the FANTOM6 project generates annotations of gene products, including large amounts of genome-independent information (4–6). We created ZENBU-Reports to enable scientists to build websites to display and disseminate their genomic and genome-independent data with minimal programming and hardware demands. ZENBU-Reports is a scientific data visualization and web-portal building web application that uses graphical interfaces to build web pages while providing secure data management and storage facilities.

## MATERIALS AND METHODS

**Figure 1.**
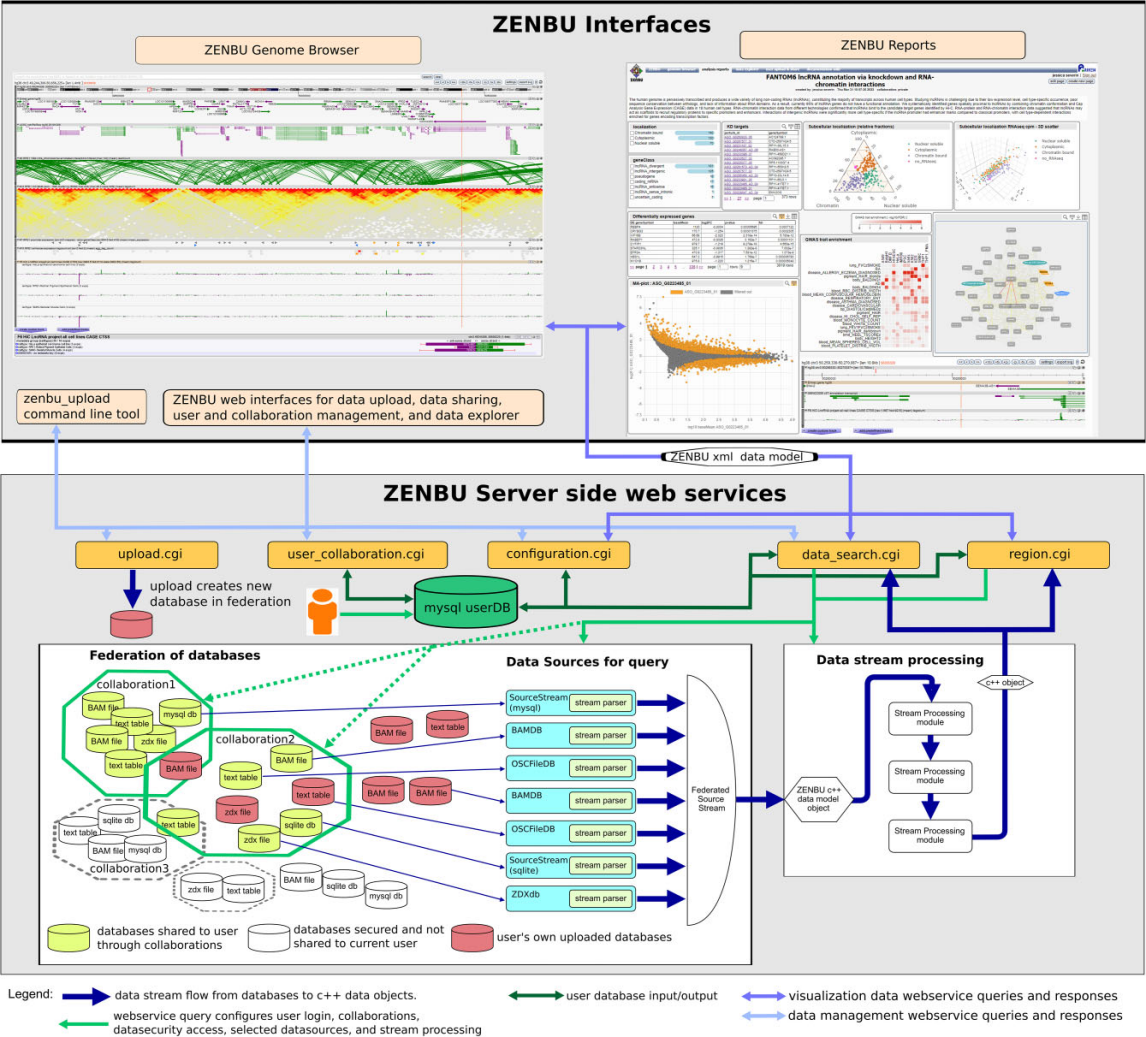
Overview of the relationship between ZENBU Reports (top right), the ZENBU genome browser (top left) and the ZENBU server side (bottom) with web services, federated database, data upload, and secured collaboration data management system.

ZENBU-Reports is a Web 2.0 style web application consisting of a server and a client (Figure 1). The client runs in the visitor's web browser and obtains data from the server through web services, taking advantage of the ZENBU server and data management infrastructure for user uploaded data, secured collaborations for data sharing, and fast data access within massive datasets (Figure 1). ZENBU-Reports is open source and can be installed on a local server if one chooses not to take advantage of the RIKEN server infrastructure.

### Server side infrastructure

Server side code is written in C++ to optimize speed performance and minimize memory usage. ZENBU (1) uses a flexible data model and an on-demand stream-processing design architecture (Figure 1). In ZENBU-Reports, we created a novel data storage and indexing system to efficiently store and retrieve paired data such as Hi-C interaction maps, network graphs, lists of differentially expressed genes, and gene enrichment analysis results, which can be joined together into complex data hierarchies providing on-demand data loading capabilities. Taking advantage of the existing data previously uploaded into the ZENBU genome browser, we provide an integrated environment to visualize genomics, tabular, and interaction data.

### Client-side code

The visualization and interface code is written in object-oriented JavaScript that renders web pages with HTML, SVG or by utilizing graphical toolkits where appropriate. Selection, filters, and searches on loaded data are managed in the web browser for immediate updates to the web page, while event-triggers allow for on-demand data loading. Data are queried from the server, and can be shared between Elements on a page. Following the ZENBU data model and functions (1), all Elements inherit from a single superclass to simplify extending existing Elements or adding new Element types in the future.

### Superclass shared functionality

The superclass queries the server through the ZENBU web services, parses its response, and stores the data as objects in arrays with various tagged metadata. Dynamic data loading, data processing, and visualization is provided by triggers, which include interaction events like selecting a data point or row, as well as system notifications generated for example upon completion of specific tasks. The trigger system is configured via a graphical interface with predefined pull-down lists to specify which events cause the trigger, which target Element the trigger will notify, and what action should be performed by the target Element. Triggers can be cascaded to allow for complex behaviors.

The data sources and the overall appearance of the Element can be specified through a core configuration panel. In addition, a common set of sub-panels allows for interaction with the loaded data:

- a data columns panel shows the different column/attribute types of the returned data objects and provides an interface for choosing which columns are shown by default and which columns visitors are allowed to show or hide, for changing the column order, and for renaming columns;
- a search panel for text searching, supporting the logical operators AND, OR, NOT, and search term grouping that are available throughout ZENBU;
- a data filter panel for applying min/max limits on numerical signal columns;
- a data download panel for returning the filtered results.

Page layout algorithms allow for Elements to be placed at fixed locations on the page or nested into Layout Elements of rows, columns or tab structures to create complex layouts, allowing for configuration of left/right or top/bottom justification depending on context. The page layout system provides a user-friendly drag-drop and snap-to-location interface to place elements on the page.

### Subclass specific visualization and functionality

Element subclasses implement the specific configuration panels, as well as functions and any additional data processing required for visualization. Subclasses were implemented using existing toolkits when possible, and newly coded otherwise.

**Figure 2.**
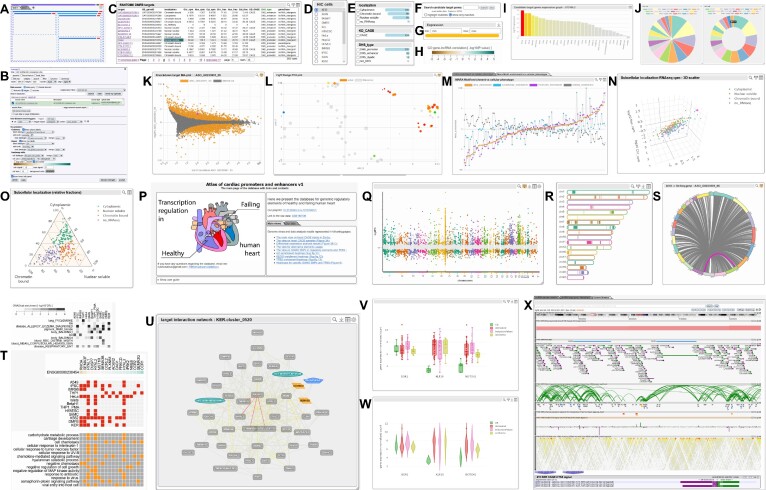
ZENBU-Reports provides user-configurable Elements that are displayed on a web page using a graphical page-layout system of nested rows, columns and tabs (**A**). Each Element is associated with a set of dynamically loaded data sources. Elements can interact with each other via event triggers, and data sources can be shared with other Elements. Elements are configurable through a configuration panel (**B**). The primary Element type is a Table (**C**). Selecting a row in the table can trigger events to other Elements. A TreeList Element (**D**) is a simplified table. The Category Filter (**E**) allows data sets to be filtered. Search (**F**) and Filter (**G**) interfaces can be displayed or hidden. The Color Scale (**H**) Element shows a legend scale for a linked graphical Element of colored items. Bar graphs(**I**), Pie and Donut charts (**J**), 2D scatter plots (**K**), Bubble plots (**L**), Line graphs (**M**), interactive 3D scatter plots (**N**), and Ternary plots (**O**) are provided. A general purpose HTML Element (**P**) allows for direct input of HTML code for static text, images, hyperlinks or other page styling. Genome-wide Elements include a Manhattan Plot (**Q**), Chromosome view (**R**), and Circos-like circular graph showing connections between genomic regions (**S**). A Heatmap Element (**T**) can represent either color-scaled or binary data. An interactive Cytoscape (7) Element (**U**) visualizes networks. Box plots (**V**) and Violin plots (**W**) compare statistical distributions. ZENBU genome browser (**X**) instances can be embedded as Elements on a page and can interact with and be controlled by other Elements on the page through event triggers. The diagram in Panel (P) was modified from an image obtained from Wikimedia Commons under the Creative Commons Attribution-Share Alike 3.0 Unported license.

Element subclasses currently in the system are listed below, with their visualization types displayed in Figure 2.

The **Table Element** subclass generates HTML code to visualize data in a table format. The table can be displayed in either a paging style or a scrollable window. In addition to the functionality provided by the superclass, the table can be sorted by clicking on column headers. Coloring options and configurable hover-over features are available.

The **TreeList Element** subclass generates HTML code to show a scrollable display of simple lists, nested lists in a tree structure, or radio-button lists as a single column.

The **HTML Element** subclass provides a space to insert and display any static HTML content, while allowing access to a data object selected in another Element sharing its data.

The **Chart Element** subclass presents general-purpose charts, graphs, and plots. It takes advantage of the chart.js toolkit (https://www.chartjs.org/) for drawing line, bar, 2d scatter, and bubble charts; and the plotly JavaScript toolkit (https://plotly.com/javascript/) for displaying 3d scatter, ternary plots, box plots, and violin plots. Different configuration interfaces are provided as appropriate for each figure type.

The **Category Element** subclass calculates a histogram of the values in a specified column from a shared Element's loaded data, and draws it as a filter list rendered in HTML, or a pie chart, donut chart, or bar chart implemented using chart.js. A filter list includes checkboxes to allow visitors to select categories for filtering, and may visualize the frequency of each category as a horizontal bar chart. Multiple Category Elements can be attached to a common shared datasource (for example a Table) and be used as AND or OR logical filters. A select-all/select-none button is provided for convenience. After selecting all categories, users may deselect categories that are to be excluded, thereby effectively creating a NOT logical filter.

The **Genomewide Element** subclass generates SVG code to visualize genomic location data across the entire genome either as a Manhattan plot or as a chromosome view. A Manhattan plot shows chromosome location along the x-axis and numerical signal values on the y-axis. Points can be colored either by chromosome, or by their value on the y-axis or from any other data column, mapped to a color scale. In a chromosome view, each chromosome is drawn as a horizontal bar proportional to the chromosome size. Within each bar, genomic location data is drawn as thin vertical lines colored either by chromosome or by their value from any other data column, mapped to a color scale.

The **Heatmap Element** subclass generates SVG code to draw a heatmap, with rows and columns corresponding to two columns in a data table, while the heatmap cell value is taken from a third column in the table. The color of each cell is determined by its value mapped to a color scale. Multiple heatmaps can thus be derived from a single table by selecting different columns for display. Columns and rows of a heatmap can be independently sorted by column or row label, by a numerical column within the dataset, or by clustering based on the values displayed in the heatmap.

The **Cytoscape Element** subclass uses the Cytoscape.js (https://js.cytoscape.org/) tool kit (7) to show a fully functional Cytoscape network graph. Data columns can be selected in the configuration panel to dynamically specify node shape, node color, and edge color.

The **ZENBU genome browser Element** subclass is a wrapper for a ZENBU genome browser instance. Multiple ZENBU genome browser instances can be included in the same ZENBU-Reports page. Elements on the page can control the genome browser by event-triggers, and genome browser data can be shared with other page Elements.

The **Circos Element** subclass generates SVG to draw a simplified Circos-like graph showing chromosomes around the edge of a circle, and interactions between genomic regions as arcs through the circle. The colors of the chromosomes and arcs are configurable.

The **ColorScale** subclass generates SVG code to display a configurable color spectrum scale with numerical range markers and legends. The scale can either dynamically adjust to the currently loaded data, or be set to a fixed range.

The **Filter Element** subclass generates HTML code to provide an interactive interface to the signal filter system, displayed in the foreground of the page. Changes to the filter trigger updates to Elements associated with the filter.

The **Search Element** subclass generates HTML code to provide an interactive search interface for data within a shared Element, displayed in the foreground of the page. Search Elements support the logical operators AND, OR, and NOT, as well as search term grouping.

The **Layout Element** subclass generates SVG code to draw the layout while editing a page, and stores the configuration parameters used by the page layout algorithm.

## RESULTS

ZENBU-Reports incorporates design concepts from wiki-pages, google-sheets/docs, and page-layout programs, and lets users create interactive scientific web pages by dragging and dropping data driven visualization Elements onto pages and configuring them with graphical interfaces. ZENBU-Reports provides the graphical Elements (exhibited in Figure 2) commonly used in article figures and supplementary websites.

The layout of a ZENBU-Reports page consists of nestable rows, columns, and tab structures. Each Element on a page is associated with data sources that can query a selected set of uploaded data. Data can be shared between Elements, which enables interactivity while reducing the amount of data transfers needed as well as the load on the server. The Elements on a page can be linked to each other with event triggers, allowing dynamic updates to the data presented.

Pages created by a user and data uploaded into ZENBU can be secured and shared only with user-specified collaborations of colleagues. The ZENBU server hardware at RIKEN is self-contained with local data storage, and accessible only to system administrators, providing security and isolation. The web portal can be saved to a permanent URL for sharing as a reference in scientific publications. Upon publication, web portal pages and data can be opened to the public, allowing any visitor to access and interact with the pages and data.

The following four example pages demonstrate the concept and breadth of ZENBU-Reports:

**Example 1**: https://fantom.gsc.riken.jp/zenbu/reports/#heart%20CAGE%20DE

This page, created from the Atlas of Heart Promoters and Enhancers (8), shows genes differentially expressed between atrium and ventricle (Supplemental Figure S1). Genes are listed in a Table and visualized in a linked 2D scatter plot. Category filters can refine the data. Tabs allow for switching from Atrium to Ventricle. A ZENBU genome browser Element displays gene locus, genomic annotations and CAGE expression tracks. The annotation table, the scatter plot, and genomic tracks are connected through an event trigger to highlight shared elements selected by the visitor. Selection of a gene or point in the plot will navigate visitors to the gene's position in the ZENBU genome browser.

To demonstrate how this page may be used in practice, we focus on enhancers supported by both ‘consensus_regions_class’ and ‘chip_class’ by selecting those category filters, and hide the ‘filtered out‘ regions in the scatter plot chart. We click in turn on each of the 14 remaining points in the scatter plot, triggering the genome browser to update to the genomic location of the region corresponding to the selected point. When we find the enhancer region overlapping the TRAJ49 gene, we zoom out on the genome browser to inspect the genomic neighborhood, and find in the CAGE transcriptome tracks that this enhancer is highly expressed only in the healthy heart atrium.

A video demonstrating how to build a simplified version of this page is provided here: https://zenbu-wiki.gsc.riken.jp/zenbu/wiki/index.php/ZENBU-Reports_demo_building_page.

**Example 2**: https://fantom.gsc.riken.jp/zenbu/reports/#FANTOM6_target_subcellular_localization

This page (Supplemental Figure S2) shows subcellular localization data of lncRNAs included in FANTOM6 (4). Visitors can filter lncRNAs by their localization as well as CAGE data availability and promoter class based on DNase I hypersensitivity (DHS) analysis. Filtered results are shown in the table, as a 3D scatter plot of the expression levels in cytoplasmic, nuclear, and chromatin-associated fractions, and a ternary plot of relative expression levels. Widget subpanels are available for searching and filtering. A subcellular localization category can also be selected by clicking on it in the legend of each plot. Selecting a row in the table or point in a plot will show the corresponding gene location in the linked genome browser. This page demonstrates how two different visualization Elements can share data from the same table and visualize in different ways.

To demonstrate how this page may be used, we first exclude lowly expressed lncRNAs by applying a filter on the table of lncRNAs (‘FANTOM6 DMFB targets’) by setting the minimum CPM (counts per million) to 5. We also tick the checkbox in the ‘KD_CAGE’ panel to select lncRNAs for which CAGE data were available. We click on ‘filtered out’ in the ternary plot ‘Subcellular localization (relative fractions)’ to toggle it and only show the selected lncRNAs. Hovering the mouse over a point in the ternary plot with low cytoplasmic expression shows that it corresponds to lncRNA DNM3OS. Clicking on this point triggers the genome browser to update to the DNM3OS location. The RNASeq tracks in the genome browser confirm that DNM3OS is indeed lowly expressed in the cytoplasm. In the list of selected lncRNAs, we click on the GSEA hyperlink in the row for DNM3OS, which takes us to the ZENBU-Reports page showing the Gene Set Enrichment Analysis results for this lncRNA.

**Example 3**: https://fantom.gsc.riken.jp/zenbu/reports/#FANTOM6_GSEA_ASO

This page (Supplemental Figure S3) visualizes Gene Set Enrichment Analysis (GSEA; (9,10)) results previously calculated in FANTOM6 (4), in which long non-coding RNAs were knocked down using LNA-modified GapmeR antisense oligos (ASOs; (11)). Upon clicking on an ASO, an event trigger causes the GSEA results to be loaded into the table and displayed in the linked scatter plot, which shows the *P*-value on a logarithmic scale and the normalized enrichment score. Clicking on a specific GSEA term in the table highlights it in the scatter plot and vice versa. Visitors can further interact with the data by built-in category filters, searching for ASOs, lncRNAs, or GSEA terms, or by applying cutoff filters from Element widgets and subpanels.

To demonstrate how this page may be used, we click on the magnifying glass in the ‘FANTOM6 DMFB ASO oligos’ table and search for ‘DNM3OS’ as our gene of interest, and find two antisense oligos associated with it. Selecting the antisense oligo ASO_G0230630_AD_10 causes the corresponding GSEA results to be loaded in the ‘FANTOM6 GSEA Pathways’ table. In this table, we use the filter to apply a maximum cutoff value of 0.1 on the p-value, and click on the magnifying glass to search for the term ‘kinase’, yielding 26 pathways. Choosing ‘show only matches’ will display these 26 pathways only. The scatter plot below the table then highlights these, and shows that the normalized enrichment score (NES) is positive for all 26 pathways.

**Example 4**: https://fantom.gsc.riken.jp/zenbu/reports/#Identification_of_therapeutic_targets_in_poor_outcome _AML_patients

This page (Supplemental Figure S4) provides an interactive display of potential therapeutic targets for the treatment of Acute Myeloid Leukemia, shown as a static PCA plot in Figure 1C by Hashimoto *et al.* (12). The page shows enriched GOterms as a table and as a PCA plot, a dynamically loaded table of expressed genes for the selected GOterm, a bar graph of patient gene expression of the selected expressed gene, and a table and plots of the differentially expressed genes comparing the AML conditions FLT3 wild type (FLT3-WT) and mutated (FLT3-Mut) to normal hematopoietic stem or progenitor cells (HSPC).

To demonstrate how this page may be used, we click on ‘regulation of cell proliferation’ in the ‘Revigo’ table, which highlights the corresponding point in the PCA plot and loads the expressed genes for that GOterm into the table labeled ‘GOterm expressed genes.’ Selecting gene JAG1 in this table loads its expression data into the bar graph labeled ‘patient gene expression’, with bars colored by their condition (FLT3-WT, FLT-Mut or HSPC). The bar graph shows that JAG1 is more highly expressed in both AML conditions compared to HSPC. JAG1 is also selected in the ‘RNASeq analysis’ table and in the associated scatter plots visualizing the differential expression analysis results, showing a statistically significant positive fold change for JAG1 in FLT3-mut AML compared to HSPC.

This video demonstrates how to interact with the data on this page. https://zenbu-wiki.gsc.riken.jp/zenbu/wiki/index.php/ZENBU-Reports_AML_target_page.

ZENBU-Reports can be used for large consortium projects such as FANTOM6, and for individual publications involving smaller collaborations or individual labs. Some project examples are:

- https://fantom.gsc.riken.jp/zenbu/reports/#FANTOM_miRNA_atlas (13,14)
- https://fantom.gsc.riken.jp/zenbu/reports/#FANTOM6 (4)
- https://fantom.gsc.riken.jp/zenbu/reports/#FANTOM6_iPSC (6)
- https://fantom.gsc.riken.jp/zenbu/reports/#F6_3D_lncRNA (5)
- https://fantom.gsc.riken.jp/zenbu/reports/#Atlas%20of%20cardiac%20promoters%20and%20enhancers (8)
- https://fantom.gsc.riken.jp/zenbu/reports/#Identification_of_therapeutic_targets_in_poor_outcome _AML_patients (12)
- https://fantom.gsc.riken.jp/zenbu/reports/#tCRE.report.index (15,16)

The ZENBU web system at RIKEN https://fantom.gsc.riken.jp/zenbu is free for all to use and to upload data and build ZENBU-Report web pages or ZENBU genome browser views.

## DISCUSSION

ZENBU-Reports was developed to create interactive scientific web portals for data sharing and analysis. While other programming toolkits, like Shiny (https://shiny.posit.co/) and Plotly (https://plotly.com/), are available to build custom websites, they generally require familiarity with a programming language such as JavaScript, Python, or R. In addition, general purpose programming toolkits like d3.js (https://d3js.org/) and visualization toolkits like chart.js (https://www.chartjs.org/) typically do not provide specialized tools to display genomic data, such as the built-in genome browser in ZENBU. On the other hand, specialized toolkits like NG-Circos (https://github.com/YaCui/NG-Circos), cytoscape.js (7) (https://js.cytoscape.org/) and TogoStanza (http://togostanza.org/) are excellent at the task they were designed for, but lack general scientific visualization modules. In addition, server infrastructure is either not provided, or may require a fee for larger applications. Google Sheets (http://sheets.google.com) provides a visualization web application that is free to use and requires no programming skills, but is not specialized for visualizing genome science data or for the massive dataset sizes typical in genome science, and does not allow interactive data exploration as provided by ZENBU-Reports. By being able to visualize both genomic and non-genomic data, without requiring any programming skills to create the pages or server availability to store the data, ZENBU-Reports provides a complete solution for scientific data visualization, analysis, and dissemination during the full lifespan of a research project and after completion of the project.

## Supplementary Material

lqad075_Supplemental_File

## Data Availability

ZENBU code is available at: https://github.com/jessica-severin/ZENBU under the 3-clause BSD Software License (permanent DOI: https://doi.org/10.5281/zenodo.8213403). The ZENBU web system at RIKEN https://fantom.gsc.riken.jp/zenbu is free for all to use, upload data and build ZENBU-Report web pages or ZENBU genome browser views. Documentation for the ZENBU system is available at https://zenbu-wiki.gsc.riken.jp.

## References

[1] FANTOM Consortium Severin J. , Lizio M., Harshbarger J., Kawaji H., Daub C.O., Hayashizaki Y., Bertin N., Forrest A.R.R. Interactive visualization and analysis of large-scale sequencing datasets using ZENBU. Nat. Biotechnol. 2014; 32:217–219. 24727769 10.1038/nbt.2840

[2] FANTOM Consortium and the RIKEN PMI and CLST (DGT) Forrest A.R.R. , Kawaji H., Rehli M., Baillie J.K., de Hoon M.J.L., Haberle V., Lassmann T., Kulakovskiy I.V., Lizio M. et al. A promoter-level mammalian expression atlas. Nature. 2014; 507:462–470. 24670764 10.1038/nature13182 PMC4529748

[3] Arner E. , Daub C.O., Vitting-Seerup K., Andersson R., Lilje B., Drabløs F., Lennartsson A., Rönnerblad M., Hrydziuszko O., Vitezic M. et al. Transcribed enhancers lead waves of coordinated transcription in transitioning mammalian cells. Science. 2015; 347:1010–1014. 25678556 10.1126/science.1259418 PMC4681433

[4] Ramilowski J.A. , Yip C.W., Agrawal S., Chang J.-C., Ciani Y., Kulakovskiy I.V., Mendez M., Ooi J.L.C., Ouyang J.F., Parkinson N. et al. Functional annotation of human long noncoding rnas via molecular phenotyping. Genome Res. 2020; 30:1060–1072. 32718982 10.1101/gr.254219.119 PMC7397864

[5] Agrawal S. , Alam T., Koido M., Kulakovskiy I.V., Severin J., Abugessaisa I., Buyan A., Dostie J., Itoh M., Kondo N. et al. Functional annotation of human long noncoding rnas using chromatin conformation data. 2021; bioRxiv doi:14 January 2021, preprint: not peer reviewed 10.1101/2021.01.13.426305.

[6] Yip C.W. , Hon C.-C., Yasuzawa K., Sivaraman D.M., Ramilowski J.A., Shibayama Y., Agrawal S., Prabhu A.V., Parr C., Severin J. et al. Antisense-oligonucleotide-mediated perturbation of long non-coding RNA reveals functional features in stem cells and across cell types. Cell Rep. 2022; 41:111893. 36577377 10.1016/j.celrep.2022.111893

[7] Franz M. , Lopes C.T., Huck G., Dong Y., Sumer O., Bader G.D. Cytoscape.Js: a graph theory library for visualisation and analysis. Bioinformatics. 2016; 32:309–311. 26415722 10.1093/bioinformatics/btv557 PMC4708103

[8] Deviatiiarov R.M. , Gams A., Kulakovskiy I.V., Buyan A., Meshcheryakov G., Syunyaev R., Singh R., Shah P., Tatarinova T.V., Gusev O. et al. An atlas of transcribed human cardiac promoters and enhancers reveals an important role of regulatory elements in heart failure. Nat Cardiovasc Res. 2023; 2:58–75. 10.1038/s44161-022-00182-x 39196209

[9] Subramanian A. , Tamayo P., Mootha V.K., Mukherjee S., Ebert B.L., Gillette M.A., Paulovich A., Pomeroy S.L., Golub T.R., Lander E.S. et al. Gene set enrichment analysis: a knowledge-based approach for interpreting genome-wide expression profiles. Proc. Natl. Acad. Sci. U.S.A. 2005; 102:15545–15550. 16199517 10.1073/pnas.0506580102 PMC1239896

[10] Mootha V.K. , Lindgren C.M., Eriksson K.-F., Subramanian A., Sihag S., Lehar J., Puigserver P., Carlsson E., Ridderstråle M., Laurila E. et al. PGC-1alpha-responsive genes involved in oxidative phosphorylation are coordinately downregulated in human diabetes. Nat. Genet. 2003; 34:267–273. 12808457 10.1038/ng1180

[11] Roux B.T. , Lindsay M.A., Heward J.A. Knockdown of nuclear-located enhancer rnas and long ncRNAs using locked nucleic acid GapmeRs. Methods Mol. Biol. 2017; 1468:11–18. 27662866 10.1007/978-1-4939-4035-6_2

[12] Hashimoto M. , Saito Y., Nakagawa R., Ogahara I., Takagi S., Takata S., Amitani H., Endo M., Yuki H., Ramilowski J.A. et al. Combined inhibition of XIAP and BCL2 drives maximal therapeutic efficacy in genetically diverse aggressive acute myeloid leukemia. Nat Cancer. 2021; 2:340–356. 35121960 10.1038/s43018-021-00177-w

[13] de Rie D. , Abugessaisa I., Alam T., Arner E., Arner P., Ashoor H., Åström G., Babina M., Bertin N., Burroughs A.M. et al. An integrated expression atlas of miRNAs and their promoters in human and mouse. Nat. Biotechnol. 2017; 35:872–878. 28829439 10.1038/nbt.3947 PMC5767576

[14] Alam T. , Agrawal S., Severin J., Young R.S., Andersson R., Arner E., Hasegawa A., Lizio M., Ramilowski J.A., Abugessaisa I. et al. Comparative transcriptomics of primary cells in vertebrates. Genome Res. 2020; 30:951–961. 32718981 10.1101/gr.255679.119 PMC7397866

[15] Moody J. , Kouno T., Chang J.-C., Ando Y., Carninci P., Shin J.W., Hon C.-C. SCAFE: a software suite for analysis of transcribed cis-regulatory elements in single cells. Bioinformatics. 2022; 38:5126–5128. 36173306 10.1093/bioinformatics/btac644 PMC9665856

[16] Moody J. , Kouno T., Suzuki A., Shibayama Y., Terao C., Chang J.-C., Lopez-Redondo F., Yip C.W., Ando Y., Yamamoto K. et al. Profiling of transcribed cis-regulatory elements in single cells. 2021; bioRxiv doi:04 April 2021, preprint: not peer reviewed 10.1101/2021.04.04.438388.

