# Supplementary material for: ZENBU-Reports: a graphical web-portal builder for interactive visualization and dissemination of genome-scale data
Source: NAR Genom Bioinform. 2023 Aug 21;5(3):lqad075. doi: 10.1093/nargab/lqad075 (PMC10440783; doi:10.1093/nargab/lqad075)
Supplement: lqad075_Supplemental_File [file lqad075_supplemental_file.pdf]

ZENBU-Reports: Supplemental figures

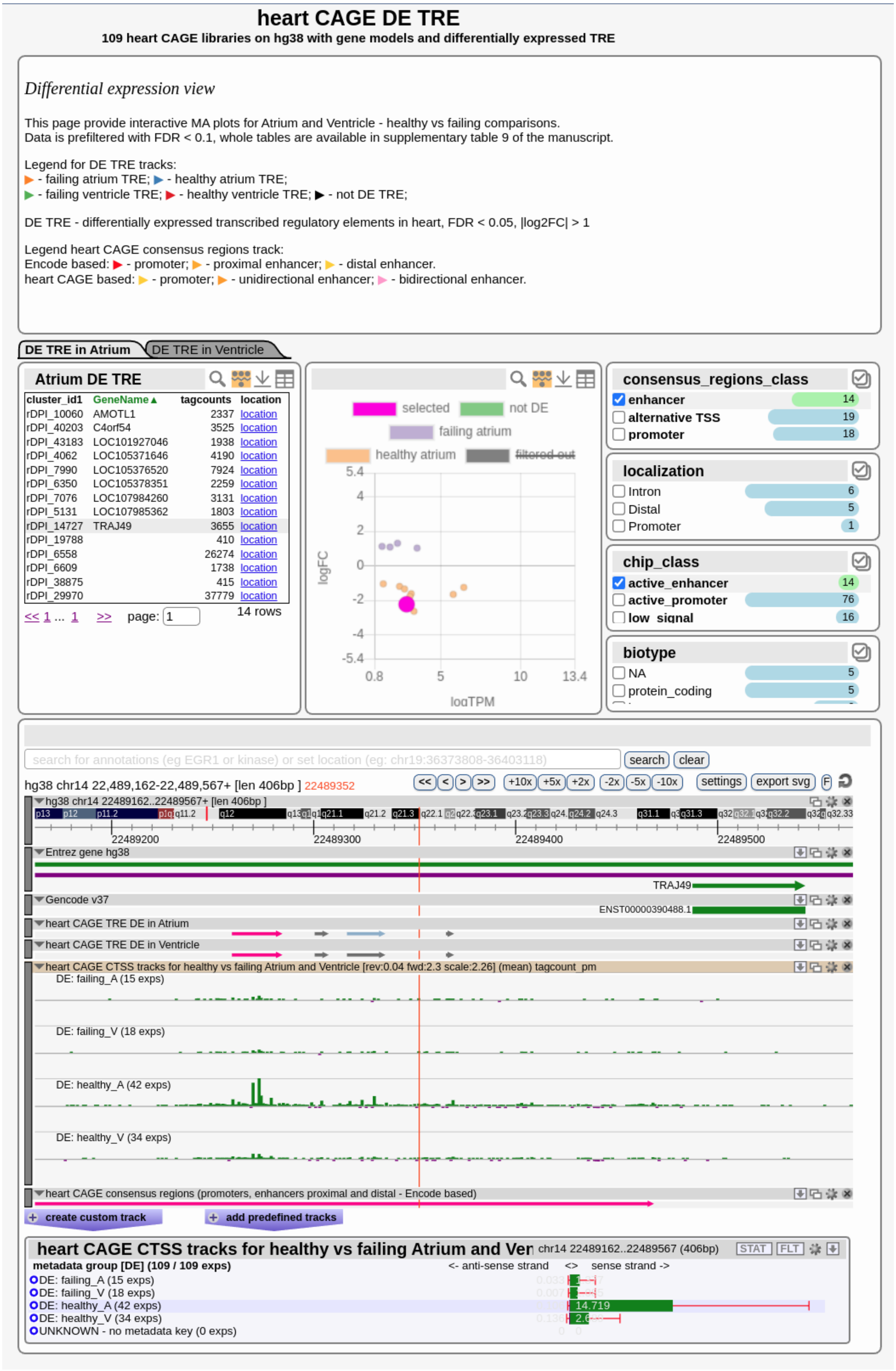

Supplemental Figure 1. ZENBU-Reports page from the Atlas of Heart Promoters and Enhancers (1).

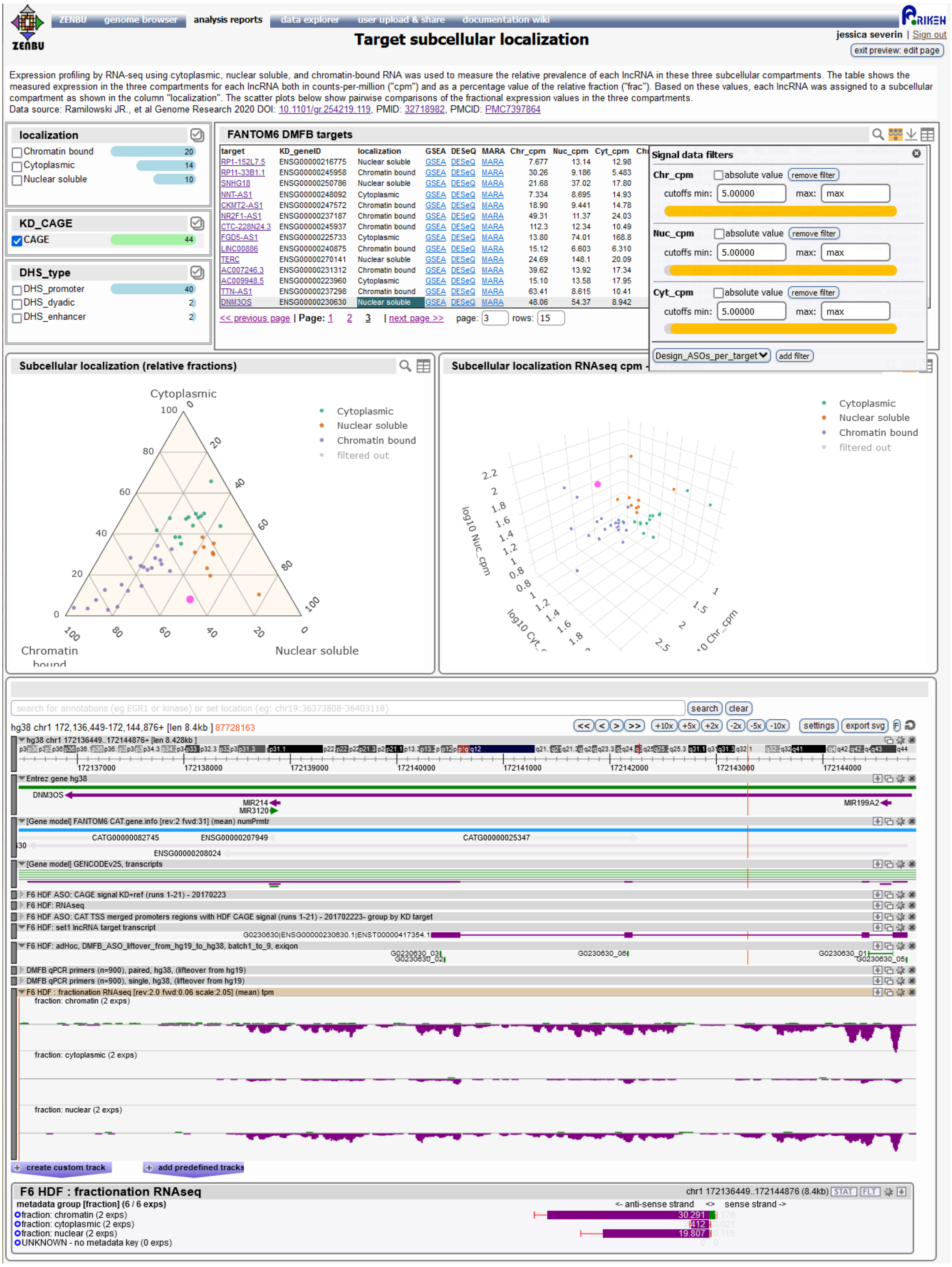

Supplemental Figure 2. ZENBU-Reports page showing subcellular localization data of lncRNAs included in FANTOM6 (2).

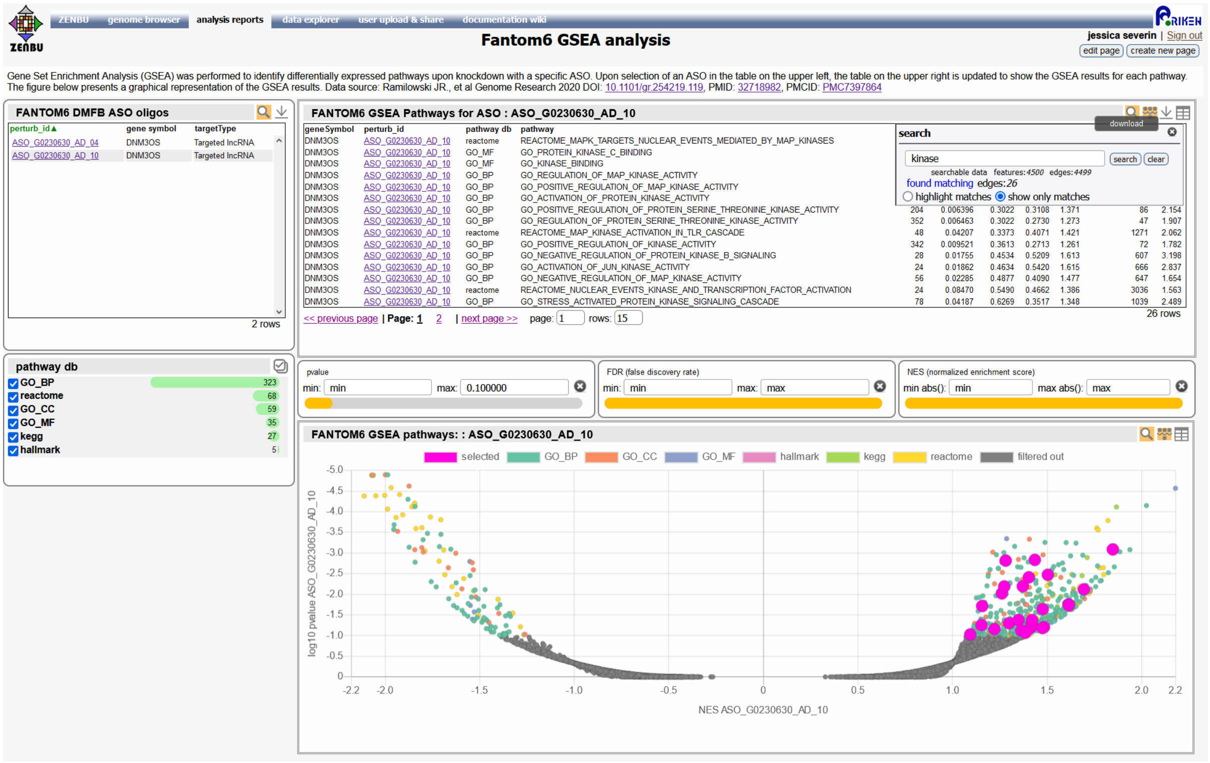

Supplemental Figure 3. Gene Set Enrichment Analysis results obtained in FANTOM6 (2),

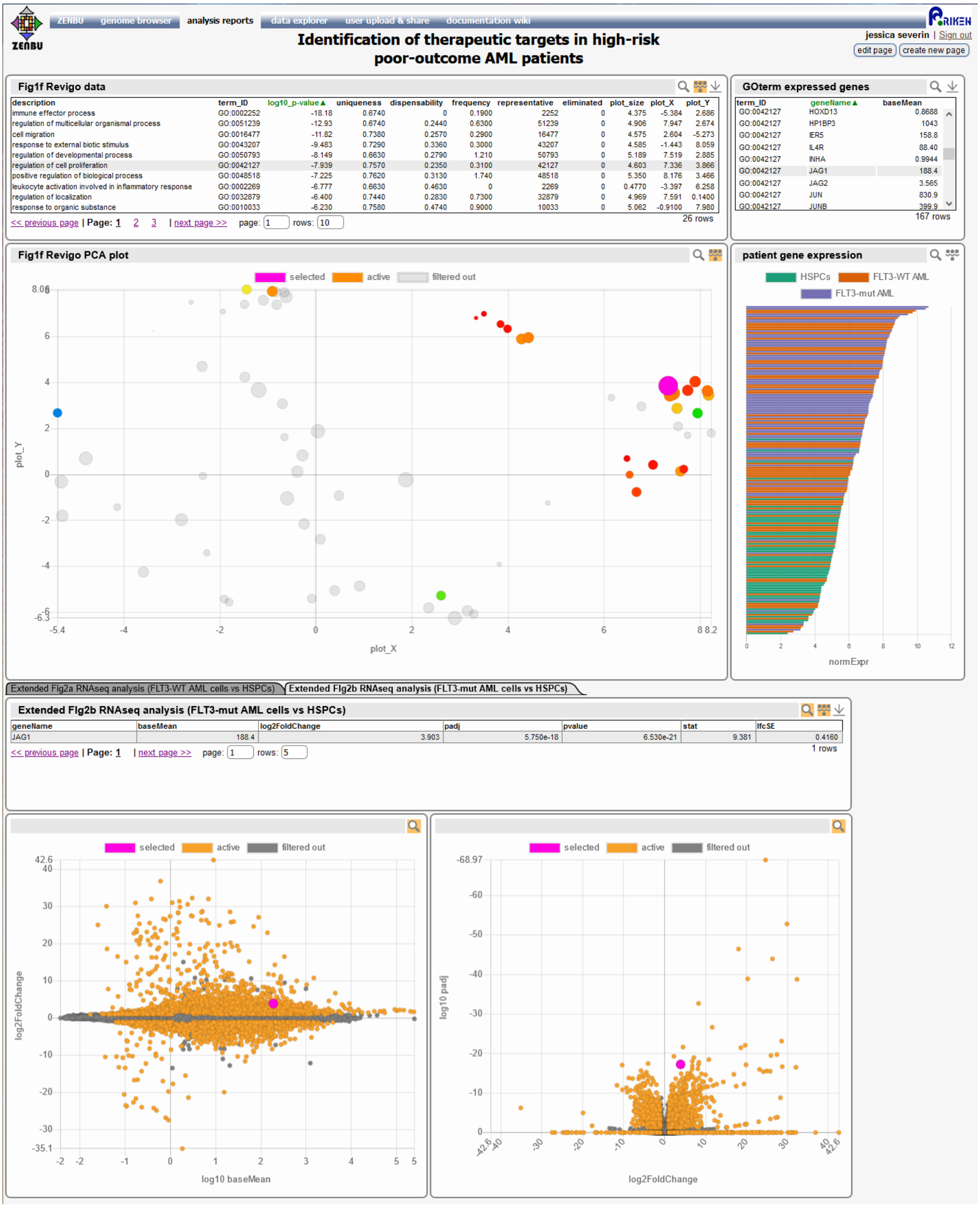

Supplemental Figure 4. ZENBU-Reports page providing an interactive PCA plot showing potential therapeutic targets for the treatment of Acute Myeloid Leukemia (3).

Bibliography

1. Deviatiiarov,R.M., Gams,A., Kulakovskiy,I.V., Buyan,A., Meshcheryakov,G., Syunyaev,R., Singh,R., Shah,P., Tatarinova,T.V., Gusev,O., *et al.* (2023) An atlas of transcribed human cardiac promoters and enhancers reveals an important role of regulatory elements in heart failure. *Nat. Cardiovasc. Res.*, doi: 10.1038/s44161-022-00182-x.

2. Ramilowski,J.A., Yip,C.W., Agrawal,S., Chang,J.-C., Ciani,Y., Kulakovskiy,I.V., Mendez,M., Ooi,J.L.C., Ouyang,J.F., Parkinson,N., *et al.* (2020) Functional annotation of human long noncoding RNAs via molecular phenotyping. *Genome Res.*, **30**, 1060–1072.

3. Hashimoto,M., Saito,Y., Nakagawa,R., Ogahara,I., Takagi,S., Takata,S., Amitani,H., Endo,M., Yuki,H., Ramilowski,J.A., *et al.* (2021) Combined inhibition of XIAP and BCL2 drives maximal therapeutic efficacy in genetically diverse aggressive acute myeloid leukemia. *Nat. Cancer*, **2**, 340–356.
